# Oxidative Stress in End-Stage Renal Disease: Pathophysiology and Potential Interventions

**DOI:** 10.1155/2023/9870138

**Published:** 2023-07-05

**Authors:** Stefanos Roumeliotis, Vassilios Liakopoulos, Evangelia Dounousi, Patrick B. Mark

**Affiliations:** ^1^Division of Nephrology and Hypertension, 1st Department of Internal Medicine, AHEPA Hospital, School of Medicine, Aristotle University of Thessaloniki, 54636 Thessaloniki, Greece; ^2^Department of Nephrology, School of Medicine, University of Ioannina, Ioannina, Greece; ^3^Institute of Cardiovascular and Molecular Sciences, Glasgow University, Glasgow, UK

Chronic kidney disease (CKD) is a major public health problem worldwide with continuously growing epidemic characteristics and heavy cardiovascular (CV) comorbidity. CKD and CV risk have a parallel course, and CV disease is the leading cause of death in end-stage kidney disease (ESKD) patients, accounting for about 50% of mortality [1].

During the past decades, atherosclerosis and CV disease have been associated, at least partially, with excessive overproduction of reactive oxygen species (ROS) and oxidative stress (OS) has emerged as a novel risk factor for CV mortality in CKD and ESKD patients [2]. OS occurs when the formation of ROS exceeds the buffering ability of the naturally occurring endogenous antioxidant defense mechanisms, thus resulting in injury and oxidation of cells and tissues and ultimately leading to CV disease. Overproduction and accumulation of ROS is present even at early CKD stages, progresses along with eGFR decline to ESKD, and is significantly reversed after kidney transplantation. Due to their high reactivity and ephemeral nature, direct and accurate measurement of ROS is very difficult. *Α*n alternative approach for assessing the redox status in CKD and ESKD is to measure the products resulting from protein, lipid, DNA, or carbohydrate damage caused by free radicals. Plasma protein carbonyls (PCO) might serve as reliable biomarkers of OS in CKD; since they are chemically stable with long half-life, their sampling is relatively easy, and there are several validated accurate detection methods; and moreover, PCO reflects accurately the state and degree of OS [3]. The oxidation of biomolecules by ROS starts very early in CKD, progresses in parallel with deterioration of kidney function, and is further exacerbated in ESKD. Circulating PCO levels are higher in patients with early CKD compared to healthy individuals and are gradually increased with reduction of estimated glomerular filtration rate (eGFR) [3]. Compared to predialysis CKD stage 4, ESKD patients undergoing dialysis present significantly increased OS. This is attributed to various factors. Typically, in this stage, physicians give strict dietary restrictions to dialysis patients to avoid consumption of fruits and vegetables that are rich in potassium to prevent hyperkalemia, thus resulting in a reduced intake of dietary antioxidants, vitamins, and flavonoids. Moreover, a certain amount of antioxidants (such as vitamin C and trace elements) is lost during every hemodialysis (HD) session. However, the main trigger for OS in HD is the contact of patients' blood with the bioincompatible, artificial dialysate, and membrane, resulting in activation of white blood cells and overproduction of ROS, after 10 minutes of every HD session [4]. Other HD-related factors causing overproduction of free radicals include the infusion of iron, anemia and inflammation, malfunctioning fistulae, the use of central venous catheters, and the use of heparin and erythropoietin agents [4]. In PD, where nearly all the above factors are absent, one might expect that the OS should be minimal. This is not the case at all; PD patients experience increased OS compared to predialysis CKD patients, but much less than HD patients. In PD, the main culprit for OS is the bioincompatible dialysate, which progressively damages the peritoneal membrane. Among proteins that are subjected to oxidative modification of their structure and function in dialysis patients, albumin is a well-established marker of nutritional and inflammation status and an independent predictor of all-cause mortality. Although PD patients present lower PCO levels and oxidized albumin levels than HD, it should be noted that a certain amount of serum albumin is also lost during PD procedure [3].

The clinical implications of OS in CKD are serious and cover a vast area of adverse events, including inflammation, atherosclerosis, CV disease, progression of CKD to ESKD, and death from any cause. Among these, the association of OS with inflammation and atherosclerosis is undisputed. Endothelial dysfunction (ED), the hallmark of atherosclerosis presents early in CKD, is triggered by OS and inflammation and is associated with CV mortality [5]. Since the first stage of ED is the oxidation of lipids and the formation of foam cells, it is crucial to investigate the pathophysiologic mechanisms underlying this process.

Proprotein convertase subtilisin/kexin type 9 (PCSK9), by regulating the expression of low-density lipoprotein (LDL) cholesterol receptor, is implicated in inflammation and ED of CKD patients. Dounousi et al. [6] performed a cross-sectional study enrolling 92 predialysis CKD patients (stages II–IV) and found that, although not correlated with eGFR, proteinuria, OS, and inflammation, plasma PCSK9 levels were associated with lipid parameters and ED, assessed by soluble intercellular adhesion molecule-1 levels. Moreover, treatment with statins increases circulating PCSK9 levels in this population and might be of benefit. Another enzyme that is involved in the pathogenesis and development of OS, inflammation, and ED through regulation of the cholesterol efflux and oxidative transformation of LDL cholesterol is soluble epoxide hydrolase 2 (EPHX2), a potential therapeutic target for CV disease [7]. In another prospective study including 118 diabetic kidney disease patients, we found that genetic variations of the EPHX2 gene (rs27411335 and rs11780592) were associated with increased oxidized LDL and carotid intima medium thickness and predicted all-cause mortality [8], indicating thus a possible genetic background in these populations.

Besides CV disease, OS is also implicated in the pathophysiology of CKD progression and various types of kidney diseases, including Balkan endemic nephropathy (BEN) [9]. The exact pathophysiological mechanisms underlying this chronic tubulointerstitial nephropathy disease have not yet been fully elucidated. OS, fibrosis, and inflammation are thought to play a role in the development and progression of BEN, but existing data are limited. Veljkovic et al. performed a cross-sectional study including 50 patients diagnosed with BEN and 38 healthy controls and found that, compared to controls, BEN patients exhibited significantly increased systemic lipid and protein oxidation status, assessed by plasma thiobarbituric acid reactive substances (TBARS) and advanced oxidation protein products (AOPPs), respectively [10]. However, in urine, only AOPP levels were significantly higher compared to controls; the urine local lipid oxidation state was not different among groups, probably due to the reduced urine lipid content.

During the past decade, there is accumulating evidence suggesting that OS plays a central role in the pathogenesis and development of diabetic kidney disease (DKD) [11]. However, the exact sites and mechanisms underlying this association have not yet been fully understood, mainly because most of the existing studies are experimental. In both *in vitro* (hyperglycemic kidney tubular epithelial cells) and *in vivo* (mouse and human kidney cells with DKD), mitochondrial general control of amino acid synthesis 5-like 1- (GCN5L1-) derived acetylation of the endogenous antioxidant manganese superoxide dismutase induces OS-mediated kidney injury, suggesting a potential novel pathway of DKD and a possible new therapeutic target [12]. To counterbalance the deleterious effects of OS in CKD and ESKD, the supplementation of exogenous antioxidants has been suggested, with the most promising being to-date the fat-soluble vitamin E in HD patients and the powerful scavenger N-acetylcysteine (NAC) in PD. An interesting approach to battle the oxidative burst caused by the exposure of blood to the artificial membrane was the coating of HD dialyzers with vitamin E. These vitamin E-coated membranes have been shown to increase the levels of vitamin E, suppress OS and inflammation markers, and improve anemia status in HD patients [13]. In predialysis CKD, it has been hypothesized that the disturbance of balance between antioxidants and prooxidants in favor of the latter might be a risk factor for CKD progression. Ilori et al. performed a large prospective study including 19,461 participants from the Reasons for Geographic and Racial Differences in Stroke (REGARDS) cohort study [14]. The authors calculated a score assessing oxidative balance by combining 13 popular prooxidants and antioxidants that were determined before enrollment, by using lifestyle and dietary assessment. After a median follow-up period of 3.5 years, the authors found that a higher score (which is indicative of higher levels of exogenous antioxidants) was correlated with significantly lower CKD prevalence. Therefore, it was hypothesized that exogenous administration of antioxidants might abrogate CKD progression. Among the supplements that were examined in CKD populations, bardoxolone methyl and pentoxifylline were shown to significantly protect from deterioration of the kidney function [15]; however, we need more well-designed trials examining novel and more powerful antioxidants.

In experimental prediabetic animal models, Akinnuga et al. showed that bredemolic acid improved glucose homeostasis and markers of kidney function, decreased malondialdehyde (a marker of lipid peroxidation status), and increased the levels of various antioxidants, including glutathione peroxidase, superoxide dismutase, and total antioxidant capacity [16]. The authors hypothesized that these findings might suggest a possible renoprotective effect of this agent, through its antioxidant effects, in an experimental induced prediabetic state. To further investigate new therapeutic, antioxidant strategies in DKD, Huang et al. performed a mixed *in vitro* and *in vivo* study and examined the possible beneficial effect of short fatty acid supplementation (acetate, propionate, and butyrate) in streptozotocin-induced type 2 diabetes/high-fat diet and DKD mice and in glomerular mesangial cells from high glucose-induced mouse models [17]. Administration of fatty acids, especially butyrate, decreased insulin resistance, prevented proteinuria development and eGFR decline in animals, and suppressed the hyperglycemia-derived OS in mouse glomerular cells, thus suggesting a potential renoprotective effect of short fatty acids, through improvement of OS.

Antioxidant agents may also have a role in the prevention of acute kidney injury (AKI) that may result from nephrotoxic agents or treatments, because the main pathophysiologic pathway in these cases is formation of ROS [18]. NAC has been widely used to prevent contrast-induced nephropathy, a common complication following the exposure to imaging iodinated contrast media [19]. To protect tumor patients treated with cisplatin from AKI, amifostine is usually prescribed as an add-on chemoprotective drug; however, this drug has several side effects. An experimental antioxidant agent (XH-003) has been shown to exert chemoprotective properties similar to that of amifostine, but without causing the adverse side effects of the drug. In the experimental study by Liu et al., HX-003 was shown to decrease the cisplatin-derived AKI through reduction of free radicals and upregulation of the activity of the antioxidant enzymes superoxide dismutase, catalase, and glutathione peroxidase [20].

This special issue is compatible and consistent with our attempt to elucidate the pathophysiologic mechanisms through which OS affects cells, tissues, organs, and biomolecules and its impact on health outcomes in CKD and ESKD. The increasing knowledge of the pathophysiology might provide further insights in the management of OS and in the evaluation of novel, therapeutic, antioxidant treatments that might benefit CKD and ESKD patients at the clinical level. This is an ongoing process, and we still need more evidence and data.

*Stefanos Roumeliotis* *Vassilios Liakopoulos* *Evangelia Dounousi* *Patrick B. Mark*
